# A novel AP2/ERF transcription factor, NtERF10, positively regulates plant height in tobacco

**DOI:** 10.1007/s11248-024-00383-z

**Published:** 2024-08-06

**Authors:** Li Xu, Yong Xu, Jia-rui Jiang, Chang-xin Cheng, Wen-wu Yang, Le-le Deng, Qi-li Mi, Wan-li Zeng, Jing Li, Qian Gao, Hai-ying Xiang, Xue-mei Li

**Affiliations:** 1 China Tobacco Yunnan Industrial Co., Ltd., Kunming, China; 2 Hongyun honghe Tobacco Group Co. Ltd, Kunming, China

**Keywords:** *NtERF10*, Plant height, Transcriptome, Metabolome, Tobacco

## Abstract

Ethylene response factors have been shown to be involved in the effects of plant developmental processes and to regulate stress tolerance. The aim of this study was to recognize the regulatory mechanisms of ethylene response factors on tobacco plant height. In this study, a gene-edited mutant (ERF10-KO) and wild type (WT) were utilized as experimental materials. Transcriptome and metabolome analyses were used to investigate the regulatory mechanism of *NtERF10* gene editing on plant height in tobacco. Here, through the analysis of differentially expressed genes (DEGs), 2051 genes were upregulated and 1965 genes were downregulated. We characterized the different ERF10-KO and WT plant heights and identified key genes for photosynthesis, the plant hormone signal transduction pathway and the terpene biosynthesis pathway. NtERF10 was found to affect the growth and development of tobacco by regulating the expression levels of the *PSAA*, *PSBA*, *GLY17* and *GGP3* genes. Amino acid metabolism was analyzed by combining analyses of differentially expressed genes (DEGs) and differentially accumulated metabolites (DAMs). In addition, we found that members of the bHLH, NAC, MYB, and WRKY transcription factor families have vital roles in regulating plant height. This study not only provides important insights into the positive regulation of the ethylene response factor NtERF10 on plant height during plant growth and development but also provides new research ideas for tobacco molecular breeding.

**Supplementary Information:**

The online version contains supplementary material available at 10.1007/s11248-024-00383-z.

## Introduction

The AP2/ERF (APETALA2/ethylene-responsive factor) family is one of the largest transcription factor families in the plant kingdom (Riechmann et al. 2000). The AP2/ERF members containing a single AP2/ERF DNA-binding domain can be classified into two subfamilies, the ERF and the dehydration-responsive element-binding subfamilies (Wessler 2005). The ERF members recognize GCC-box cis-acting elements (AGCCGCC) in the promoters of ethylene-responsive genes (Ohme-Takagi and Shinshi 1995), and genes in the ERF family encode transcriptional regulators with a variety of functions involved in developmental and physiological processes in plants (Nakano et al. 2006). The AP2/ERF family is a plant-specific transcription factor family whose members have been associated with various developmental processes and stress tolerance (Somayeh et al. 2018; Licausi et al. 2013; Li et al. 2015). Due to their plasticity and to the specificity of individual members of this family, AP2/ERF transcription factors represent valuable targets for genetic engineering and breeding of crops.

Transcriptional regulation can determine numerous agronomically important traits. Plant height, yield and other agronomic traits have always been the focus of plant science in crops. There is a close relationship between plant height and yield, and plant height is not only a decisive factor in plant architecture but also an important agronomic trait directly linked to yield potential (Wei et al. 2010; Wang and Li 2006). Plant height is an important agronomic trait and plays an important role in plant breeding. Numerous investigations have shown that in many species, particularly grasses and crop species, DREB1s have functions similar to those in the model plant Arabidopsis. Ectopic expression of Arabidopsis *DREB1s* in other plant species (Zhang et al. 2004; Hsieh et al. 2002) or expression of *DREB1s* from other plants in Arabidopsis (Tong et al. 2009) have been found to produce similar phenotypes, such as enhanced tolerance to abiotic stress, dwarfism and delayed development (Gilmour et al. 2004; Agarwal et al. 2017; Morran et al. 2011). Some researchers have used CRISPR/Cas9 gene editing technology to obtain high-yield Waxy maize varieties by editing the *Waxy* gene and the ARGOS8 gene (a negative regulator of the ethylene response) to obtain drought-resistant and high-yield gene-edited maize (Gao et al. 2020; Shi et al. 2017). A new indica rice strain with low cadmium accumulation was obtained by knockout of *OsNramp5* (Tang et al. 2017)*.* These results showed that the mutant material produced through gene editing could lay the foundation for subsequent studies on the function of these genes. The CRISPR/Cas9 system is used to improve crop yield, improve quality, and enhance plant biological and abiotic stress tolerance. This technology has been widely used in plant molecular biology and plant genetic engineering.

Tobacco (*Nicotiana tabacum* L*.*) is a member of the agriculturally important *Solanaceae* family and is one of the most studied higher plant species. This is because of both its economic importance and its convenience as a model plant system for research. Tobacco can be easily transformed and has a relatively short generation time (Rushton et al. 2008b; Xu et al. 2020). Plant height is an important agronomic characteristic of tobacco and is closely related to its yield and quality (Peiffer et al. 2014; Jiang et al. 2022). An in silico analysis of 1.15 million gene-space sequence reads from the tobacco nuclear genome has been completed, and a detailed analysis of more than 2500 tobacco transcription factors (TFs) has been conducted (Rushton et al. 2008a).

The objectives of this work were to analyze the plant height transcriptome and metabolome changes in tobacco plants affected by ERF gene editing. We systematically investigated the gene expression and metabolic differences between the mutant and wild-type tobacco leaves. In addition, we revealed the potential regulatory network between genes and metabolites that plays a key role in plant growth and development. The results of this study provide a theoretical basis for a better understanding of the molecular mechanisms by which ERF genes regulate plant growth and development at the transcriptome and metabolome levels.

## Materials and methods

### Plant materials and growth conditions

In this study, a flue-cured tobacco (*Nicotiana tabacum* L*.*) cultivar named Honghua Dajinyuan, known for being widely grown in Southwest China, was utilized. NtERF10 mutants were obtained by gene editing mediated by the CRISPR/Cas9 system. The target sequence was annealed and ligated to the pOREU3TR skeleton vector previously digested with BsaI. The constructed vector pOREU3TR:NtERF10 was introduced into Honghua Dajinyuan tobacco via *Agrobacterium tumefaciens* (LBA4404)-mediated transformation. Drug-resistant seedlings were obtained and screened by molecular detection, and the T1/T2 generation of self-homozygous seedlings was obtained. Homozygotes for target editing with no transgenic labels were ultimately obtained.

The mutant (ERF10-KO) and wild-type (CK) (60) plants were grown under controlled conditions in Shilin, Yunnan. During the tobacco-growing season, especially in the later stages of growth, the wild type and the mutant plants showed significant differences in plant height. During the maturation stages, leaf samples from the wild type and the mutant were collected and designated CK and ERF10-KO, respectively. Each CK and ERF10-KO sample consisted of 4 leaves (1 leaf × 4 plants), and was frozen in liquid nitrogen and stored at − 80 °C until further use. Three biological replicates (4 individual leaves/replicate) were analyzed for the CK and ERF10-KO.

### Protein structural and phylogenetic analysis

The protein structure of NtERF10 was analyzed using the online motif scan tool. The conserved domain of NtERF10 was searched with the National Center for Biotechnology Information (NCBI) BLAST CD-search using the full-length amino acid sequence of NtERF10 as a query. Multiple protein sequence alignment was performed with the National Center for Biotechnology Information (NCBI) Smart BLAST. The plant height data processing analysis used a Shapiro–Wilk test and F test to test for normality and equal variances.

### Transcriptomics analysis

#### RNA extraction and sequencing

Samples were extracted using the TIANGEN RNAprep Pure Plant Total RNA Kit. The RNA purity was measured with Nanodrop, the RNA concentration was measured with Qubit, and the RNA integrity was assessed with Agilent 2100. The quality of the samples met the requirements of library construction and sequencing.

After the qualifying samples were tested, cDNA libraries were constructed using the NEBNext® Ultra™ RNA Library Preparation Kit for Illumina Sequencing System (NEB, USA). Sequencing was performed by Novogene Co., Ltd., Beijing.

#### Transcriptomics bioinformatics analysis

Clean reads were obtained by filtering the sequencing data, mainly after removing reads containing connectors, duplicates and low sequencing quality. Based on an analysis of the comparison results obtained with the software Bowtie2, TopHat2 was used to align the transcriptome sequencing reads to genes and to identify splicing points between exons.

Differentially expressed genes (DEGs) were identified with DESeq2 to make comparisons between groups. Genes were considered differentially expressed if they met the criteria |log2FC|> 1 and Q value < 0.05. R was used to perform correlation analyses of these DEGs.

For gene set enrichment analysis (GSEA), we first calculated the Pearson correlation coefficients of each protein and its corresponding transcript and tabulated these Pearson correlation coefficients.

### Metabolomics analysis

#### Metabolomics extraction

The plant samples for metabolomics analysis were extracted with a mixture of MeOH and H_2_O (v/v = 75:25). The lyophilized samples (0.02 g) were extracted with 1.5 mL of extractant by vortexing for 6–15 s and shaking at 30 °C and 200 rpm for 1 h. The samples were then centrifuged at 14,000 rpm/min for 10 min, and 800 μL of supernatant was transferred to an injection bottle for liquid chromatography–mass spectrometry (LC‒MS) analysis.

#### Metabolomics bioinformatics analysis

Progenesis QI software was used for data processing. The peak area normalization method was used to calculate the relative content of the measured metabolites. SIMCA14.1 software was used for statistical analysis of the data. We combined the multivariate statistical analysis of the partial least squares-discriminant analysis (PLS-DA) VIP value and the univariate statistical analysis of the T test *P* value to screen significantly different metabolites among different comparison groups. The thresholds used to determine a significant difference were VIP ≥ 1 and T test *P* value < 0.05.

## Results

### Phenotype and physiological response analysis of dwarfing plants

A sequence analysis showed that NtERF10 contained the conserved sequence of the AP2/ERF family, a conserved DNA-binding domain, the AP2/ERF domain, that is 58 amino acids in size (Fig. S1A). The sequence blast for NtERF10 in relation to AP2 members from soybean and Arabidopsis (Fig. S1B) showed that the 14th and 19th amino acids of the conserved sequence were valine and glutamic, respectively, indicating that NtERF10 belongs to the DREB subfamily in the AP2/ERF family.

**Fig. 1:**
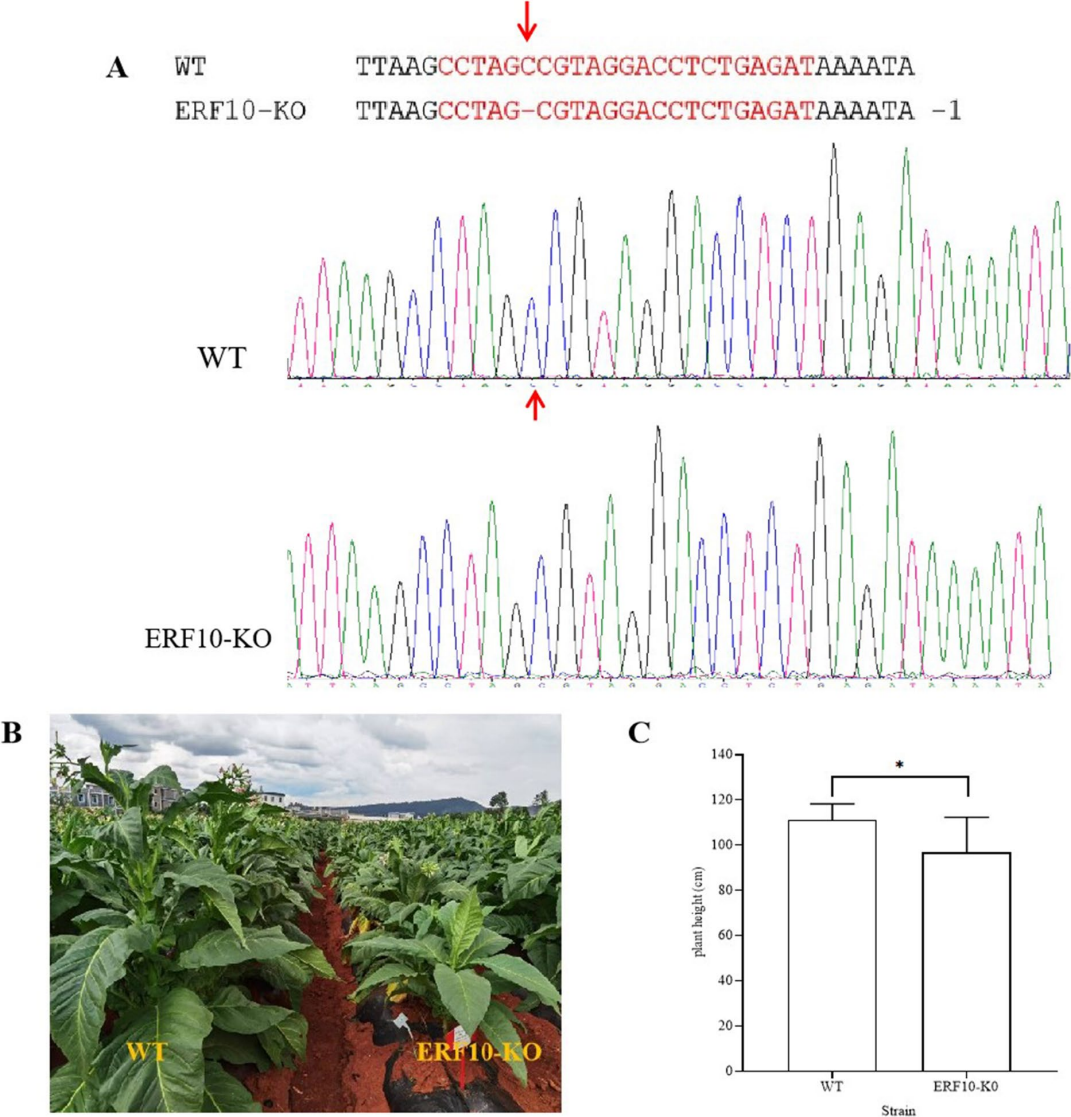
NtERF10 gene mutant lines and phenotypic analysis. **A** CRISPR/Cas9-mediated NtERF10 gene mutation in *Nicotiana tobacum *(red words indicate sgRNA sequences; position of editing is indicated by red arrow); **B** phenotype of WT and ERF10-KO mutant plants under normal conditions; **C** plant height of WT and ERF10-KO mutant plants. * and ** indicate significant differences between the results at *p* ≤ 0.05 and *p* ≤ 0.01, respectively. Bars represent the mean ± SE. (Color figure online)

CRISPR/Cas9-mediated NtERF10 knockout mutants were obtained. During the tobacco-growing season, especially in the later stages of growth, significant differences in plant height were observed between the CK (WT) and the mutant (ERF10-KO) plants. Sequencing results showed that the gene-edited lines had mutant sites: deletion of 1 base, C. The results of protein sequence alignment showed that the deletion of a base pair at the 810th base of the NtERF10 coding region caused frame shift mutation and premature termination of translation. The lines were named ERF10-KO (Fig. 1A). From the phenotype shown in Fig. 1B, it is clear that the WT plants were taller than ERF10-KO plant at the maturation stages. This result was also verified by measuring plant height at the mature stage (Fig. 1C). The results showed that compared with the height of the CK (WT) plants, the height of the *NtERF10* gene-edited mutant plants decreased under normal conditions (Fig. 1).

### Transcriptome analysis of ERF10-KO and the WT plants

**Fig. 2:**
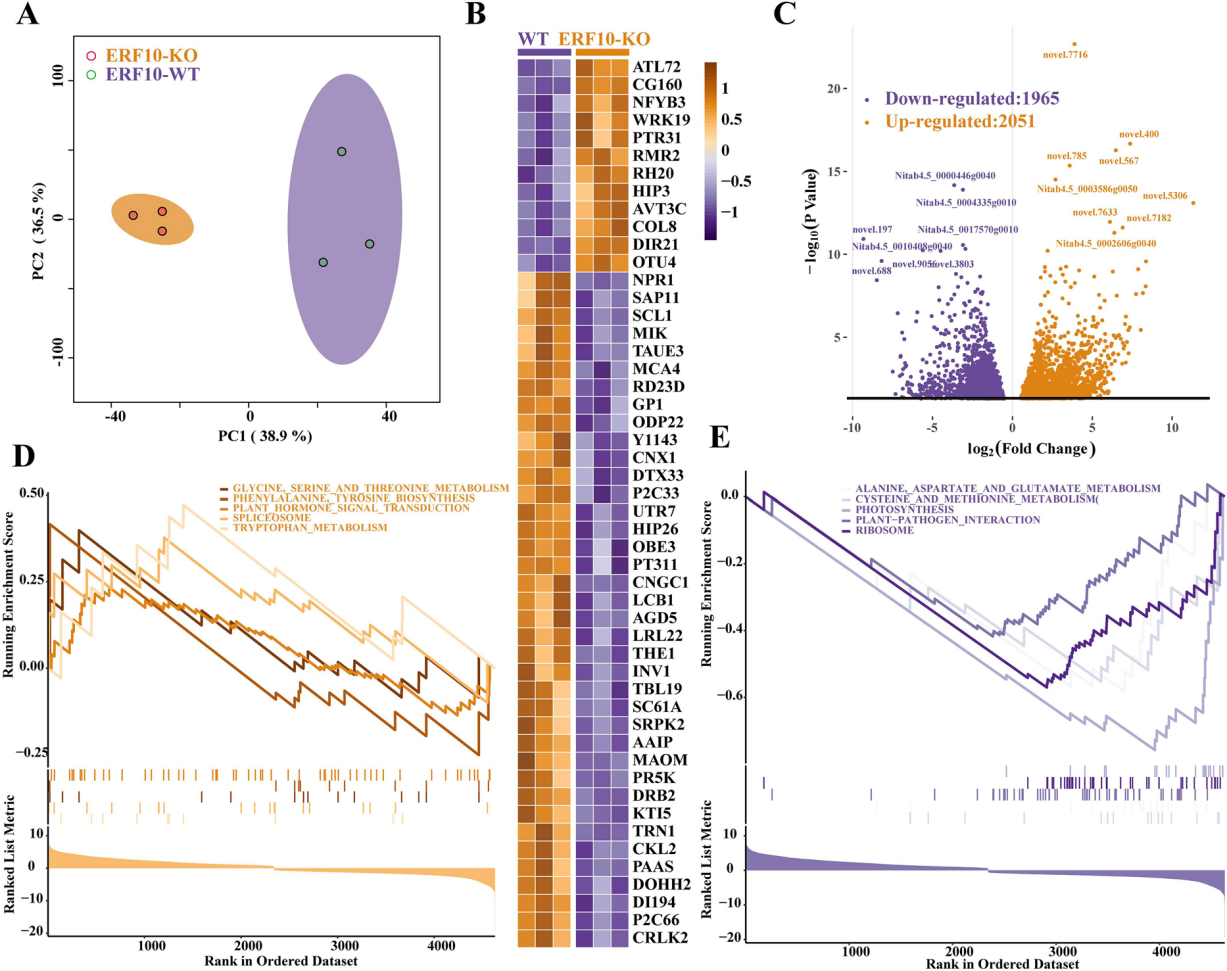
Transcriptome changes and functional analysis of tobacco dwarf mutant (ERF10-KO) and wild-type samples (WT). **A** PCA analysis of ERF10-KO and WT expression. **B** 50 genes with significant changes in ERF10-KO and WT. **C** Volcano diagram of up- and down-regulation of differential genes (DEGs). **D** GSEA enrichment analysis of five pathways that up-regulated differential gene enrichment. **E** GSEA enrichment analysis of five pathways that down-regulated differential gene enrichment

To explore the molecular mechanism of *NtERF10* gene editing in tobacco, dwarf mutant (ERF10-KO) and CK (WT) samples were selected for transcriptome analysis at the mature stage. A principal component analysis (PCA) of the samples based on fragments per kilobase of exon model per million reads mapped (FPKM) showed a clear separation of the ERF10-KO and the WT sample types, implying that the differentially accumulated metabolites between the two phenotypes are regulated by differentially expressed genes (Fig. 2A). The top 50 differentially expressed genes in the transcriptome were analyzed, and were found to mainly include transcription factors such as WRKY, growth and development and resistance-related genes NYFB, NPR, SAP, etc. (Fig. 2B).

To investigate the pivotal genes in the dwarf mutant (ERF10-KO) and the CK (WT), differentially expressed genes (DEGs) were identified according to the strict criteria of |log2-fold change (FC)|> 1 and a false discovery rate-adjusted *P* value < 0.05. A total of 4016 genes were identified, of which the expression of 2051 and 1965 was up- and downregulated, respectively (Fig. 2C). Gene set enrichment analysis (GSEA) was applied to determine the trends regarding which biological terms were overrepresented in the transcript changes in the dwarf mutants at the mature period (Fig. 2D–E). The results from the GSEA suggested that in the ERF10-KO samples, “glycine, serine and threonine metabolism”, “phenylalanine, tyrosine biosynthesis”, “plant hormone signal transduction”, “spliceosome”, “tryptophan metabolism” and related terms were overrepresented among the upregulated genes, while “alanine, aspartate and glutamate metabolism”, “cysteine and methionine metabolism”, “photosynthesis”, “plant‒pathogen interaction”, “ribosome” and related terms were overrepresented among the downregulated genes compared to the WT.

### Analysis of genes related to developmental processes and stress tolerance

**Fig. 3:**
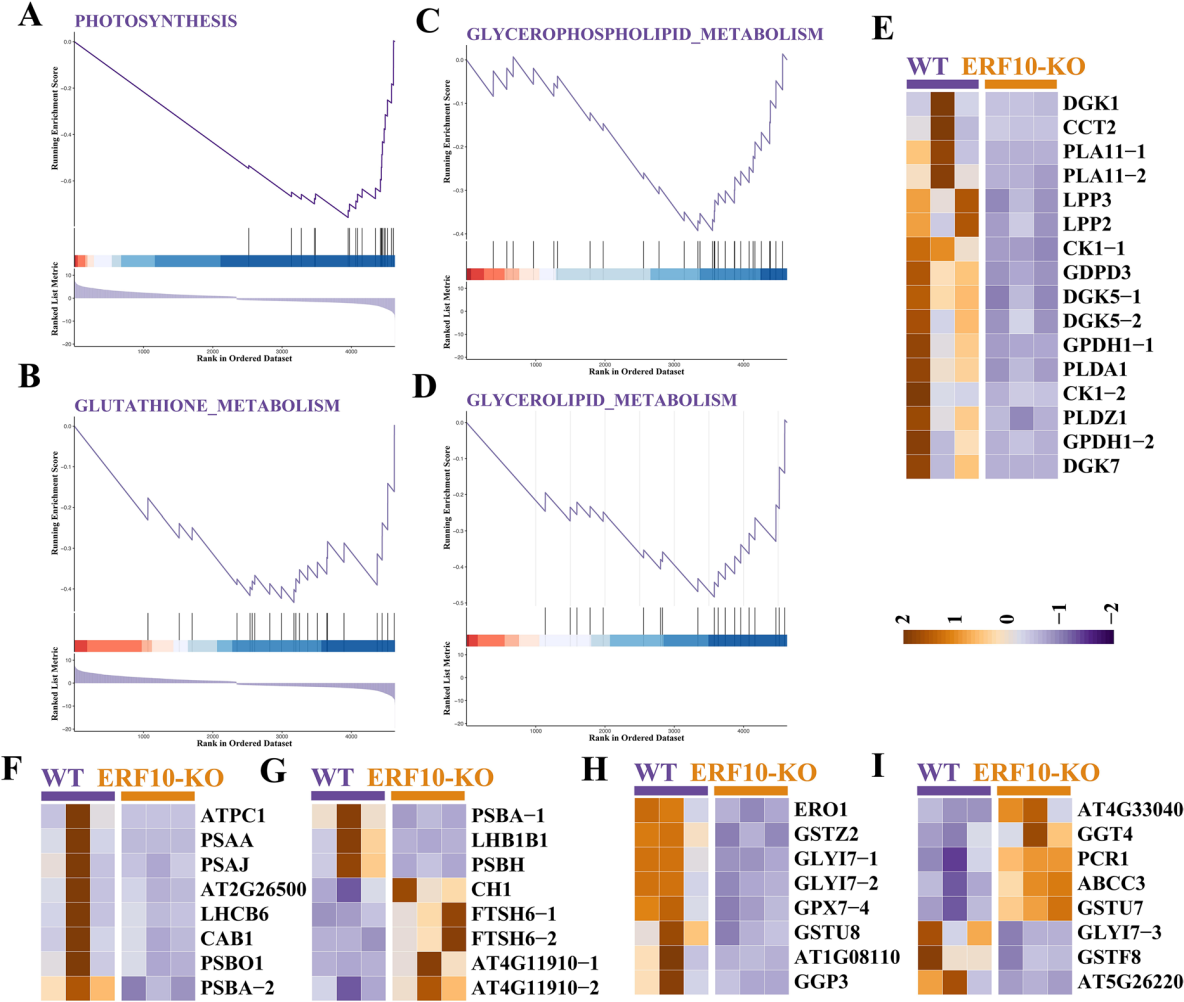
Functional study of genes that differ between ERF10-KO and WT. **A–D** GESA analyzed genes related to photosynthesis, glutathione-metabolism, glyceropholipid-metabolism, glycerolipid-metabolism. **E–I** DEGs for cell membranes lipids metabolism, photosynthesis and glutathione metabolism in ERF10-KO and WT

An analysis of the expression of differentially expressed genes in growth and development pathways between the mutant (ERF10-KO) and CK (WT) was conducted. Changes in genes associated with photosynthesis, glutathione metabolism and cell membrane lipid metabolism were observed in NtERF10 gene-edited lines (Fig. 3). The expression of cell membrane lipid metabolism-related genes such as *DGKs*, *PLAs* and *LPPs* was downregulated in ERF10 compared with the WT, and these genes were associated with plant resistance (Fig. 3E). The *PSAA* and *PSBA* genes and *GPX7-4* gene expression decreased in the photosynthetic and glutathione metabolism pathways, respectively (Fig. 3F–I). These genes play an important role in plant growth and development. *GLY17* and *GGP3* gene expression was downregulated and played a role in stress resistance.

**Fig. 4:**
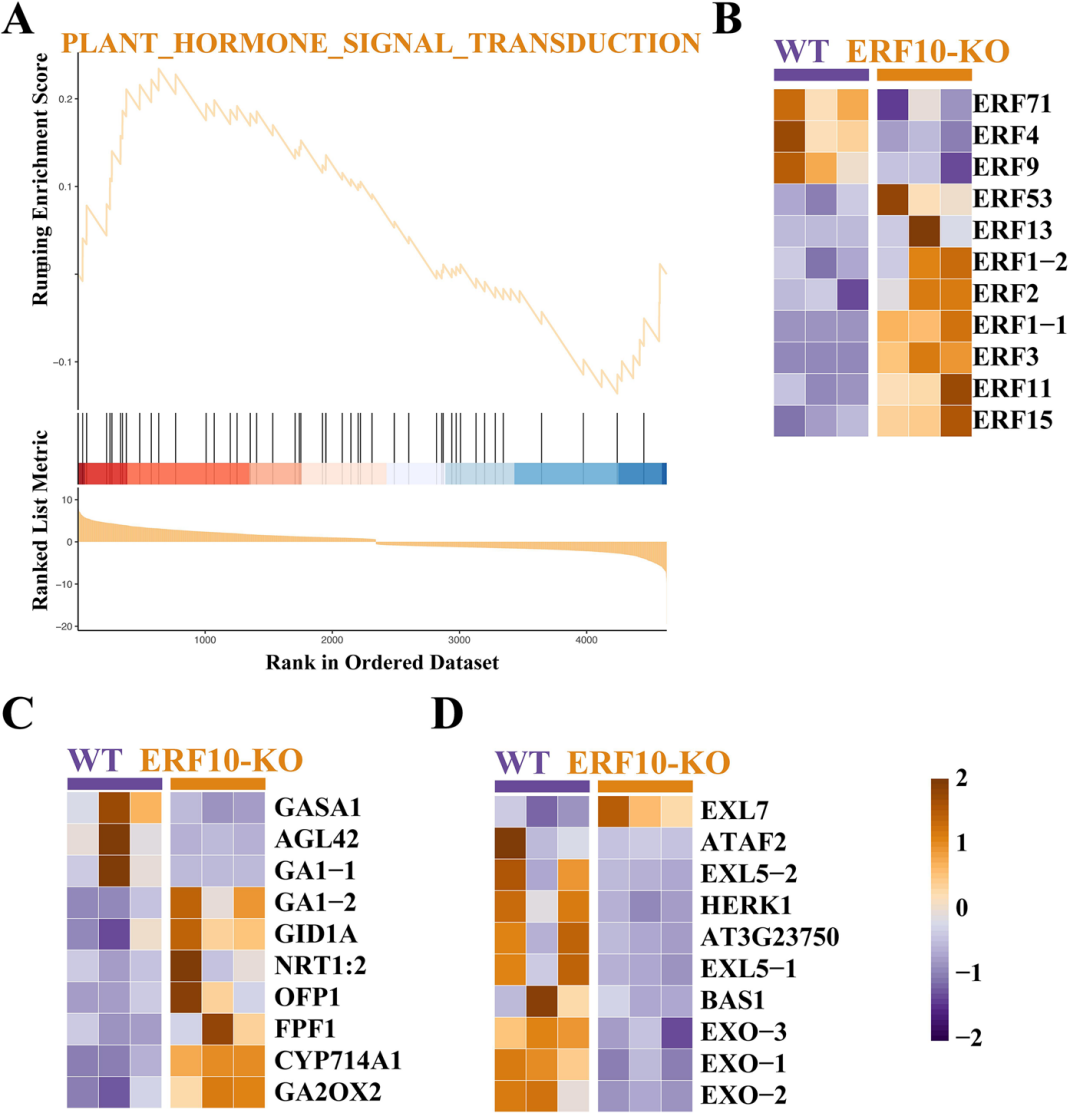
Differential genes in plant hormone-related genes. **A** GSEA analysis of plant hormone signal transduction related genes. **B–D** ethylene response, gibberellins, and Brassinosteroids production are all represented by genes

The GSEA of plant hormone signal transduction-related genes showed that the expression levels of related hormone pathways, including the ERF, GA and BR pathways, were changed in gene-edited plants (Fig. 4). The expression levels of the *ERF4*, *ERF9* and *ERF71* genes were decreased in ERF10-KO. Further investigation showed that the dwarf mutant plants had much less reduction in the expression levels of *NtERF4* than the WT*.* The expression of *GASA1, AGL42* and *GA1-1* genes related to the GA pathway was downregulated in ERF10-KO. In the BR signaling pathway, *EXO* and *EXL5* gene expression was decreased in the mutant, while *EXL7* gene expression was increased. These genes are involved in plant growth and development, stress resistance and other physiological processes.

### Expression patterns of genes related to the terpene synthesis pathway

**Fig. 5:**
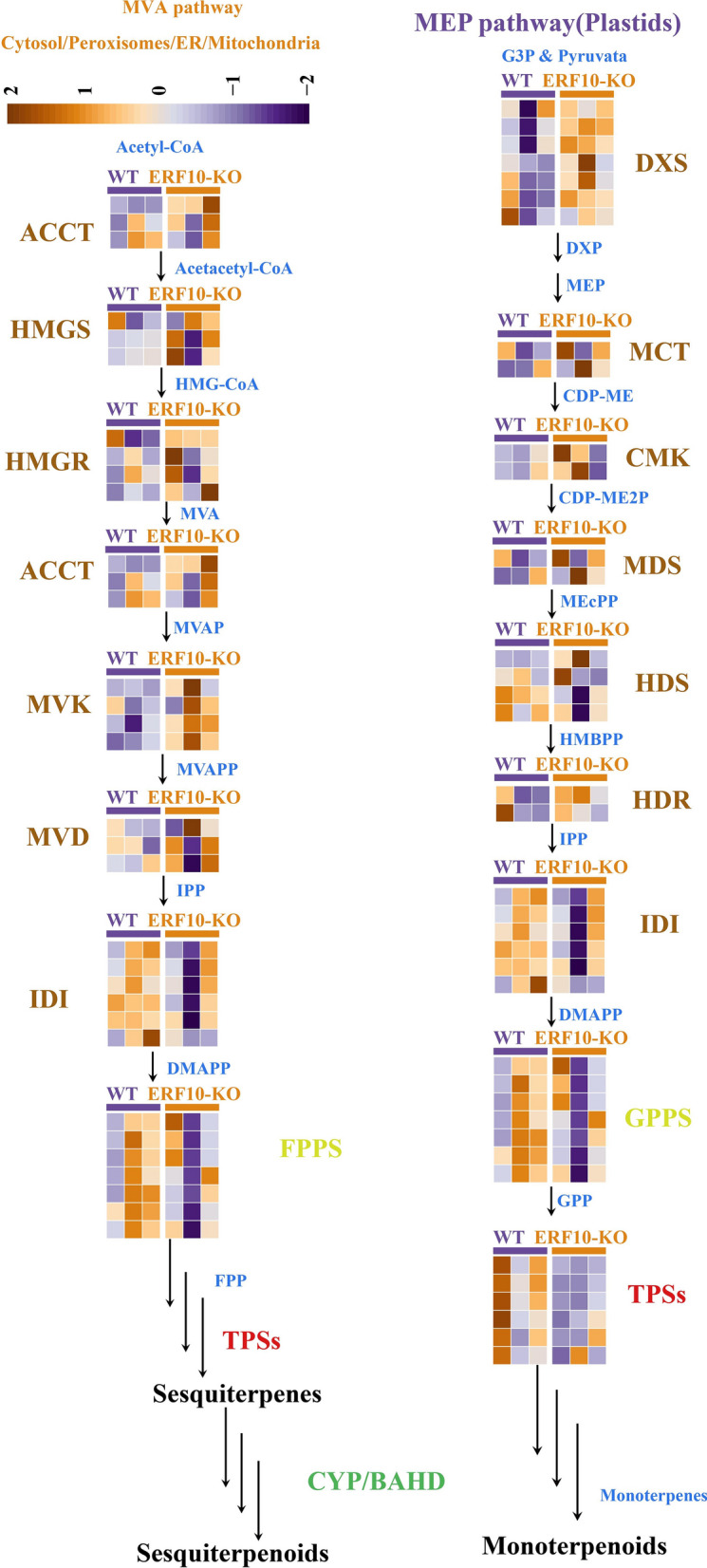
Expression patterns of terpene synthesis-related genes in ERF10-KO and WT

The terpenoid synthesis pathway is closely related to plant growth and development and the stress response. The synthesis of terpenes occurs primarily through the mevalonate (MVA) and 2-C methyl-d-erythritol-4-phosphate (MEP) pathways. To further investigate the molecular mechanisms underlying metabolite differences between ERF10-KO and WT, we analyzed the expression patterns of 70 genes related to terpene synthesis in the MVA and MEP pathways (Fig. 5). Reactions of the MVA pathway occur mainly in the cytoplasm, beginning with the condensation of acetyl-CoA. FPP can be used to synthesize sesquiterpenoids and triterpenoids through the activities of various enzymes. The MEP pathway takes place in plastids, including chloroplasts, chromoplasts, and leucoplasts. Pyruvate and glyceraldehyde-3-phosphate are the initial precursors of the MEP pathway. GPP is the biosynthetic precursor of monoterpenoids, ubiquinone, and other terpene quinones.

We measured the expression of 70 genes in the terpene biosynthesis pathway throughout the ERF10-KO and the WT plants and found that their expression patterns were quite different (Fig. 5). Some genes had low expression in the ERF10-KO plants and high expression in the WT plants (e.g., *IDI*, *FPPS*/*GPPS* and *TPSs*), whereas other genes showed the opposite pattern (e.g., *DXS*, *HMGS*, *MVK*, and *MVD*). Studies have shown that the expression levels of *IDI* and *FPPS*/*GPPS*, key genes for terpenoid synthesis, are decreased in ERF10-KO, which may be closely related to the dwarfing and poor stress resistance of ERF10-KO plants.

### Analysis of differential metabolites in dwarf mutant plants

**Fig. 6:**
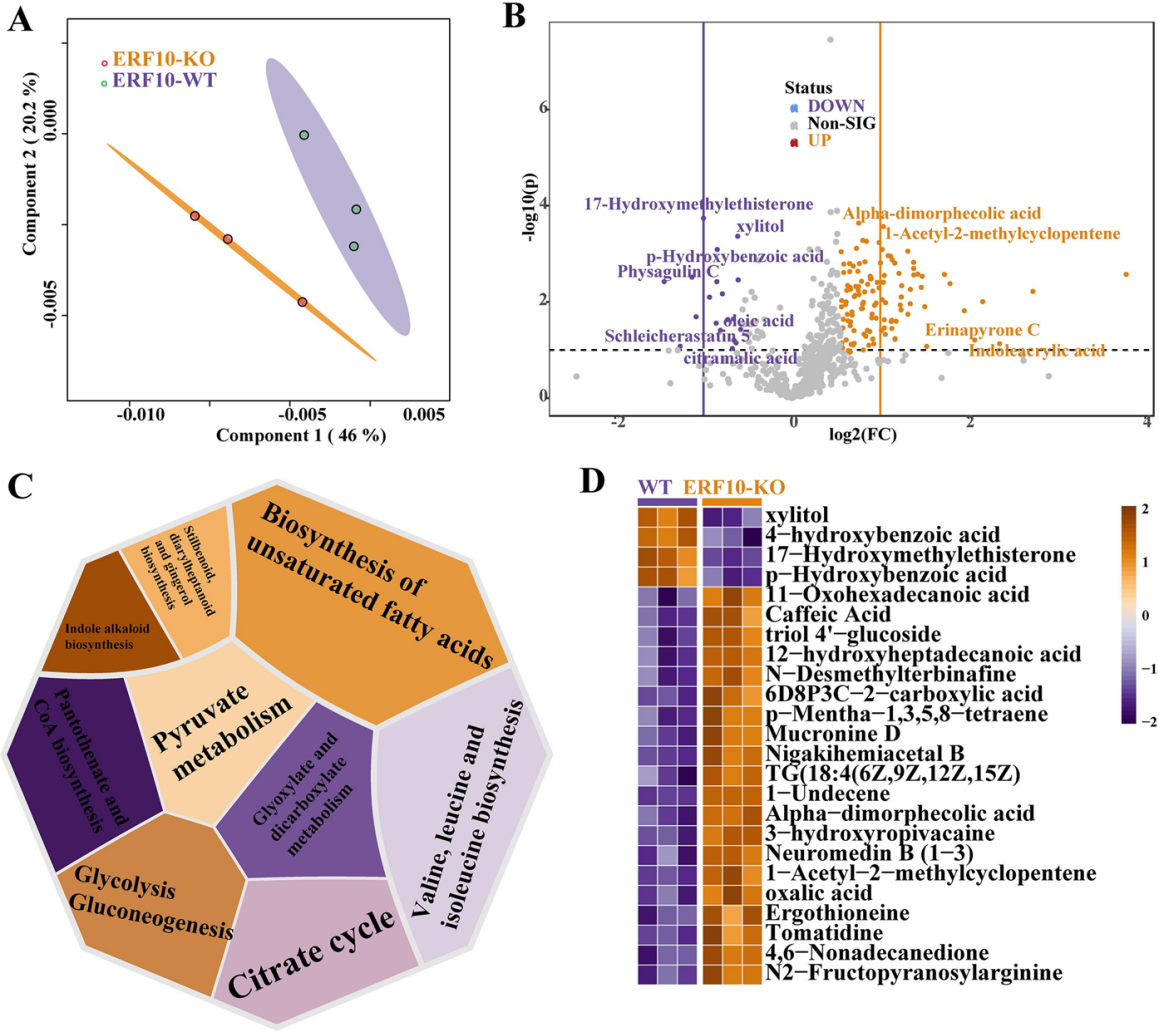
Overview of metabolome analysis between ERF10-KO and WT. **A** PLSDA analysis of metabolomic data between ERF10-KO and WT. **B** Volcano plot for metabolite differential analysis between ERF10-KO and WT. Among them, yellow represents up-regulation and purple represents down-regulation, and the differential metabolites with the most obvious changes are marked with names. **C** Pathways of major enrichment of differential metabolites. **D** Expression heatmap of top 26 differential metabolites. (Color figure online)

The metabolic profiles of the tobacco leaves of the mutant (ERF10-KO) and CK (WT) were investigated via LC‒MS (Fig. 6). Interestingly, the mutant samples (ERF10-KO) and the CK samples (WT) were clearly separated. The PCA of the metabolites showed that the metabolic patterns of ERF10-KO and the WT were significantly different. The PLS-DA model was used to screen important differentially accumulated metabolites (Fig. 6A). Based on the quantification and differential analysis of all tested metabolites. There were 128 DAMs between the ERF10-KO and the WT groups, among which 19 metabolites, such as 17-hydroxymethylethisterone, xylitol, *p*-hydroxybenzoic acid and physagulin C, were downregulated, while alpha-dimorphecolic acid, 1-acetyl-2-methylcyclopentene, Erinapyrone C and indoleacrylic acid 46 other metabolites were upregulated (Fig. 6B). Differential metabolites were mainly involved in Kyoto Encyclopedia of Genes and Genomes (KEGG) pathways. We found that the differential metabolites were mainly concentrated in primary metabolism, such as amino acid metabolism, starch and sucrose metabolism and lipid metabolism (e.g., valine, leucine and isoleucine biosynthesis, pyruvate metabolism, glycolysis gluconeogenesis, and biosynthesis of unsaturated fatty acids) (Fig. 6C). A cluster heatmap analysis of the top 25 diverse metabolites in the mutants revealed that 4-hydroxybenzoic acid, xylitol, 17-hydroxymethylet and *p*-hydroxybenzoic acid metabolites were reduced in content, while 11-oxohexadecanoic acid, caffeic acid and triol 4′-glucoside were elevated (Fig. 6D). The results indicated that the main enrichment pathways of differential metabolites in the analyzed mutants and wild type were related to plant growth and development.

### Metabolite network analysis and combined transcriptome function analysis

**Fig. 7:**
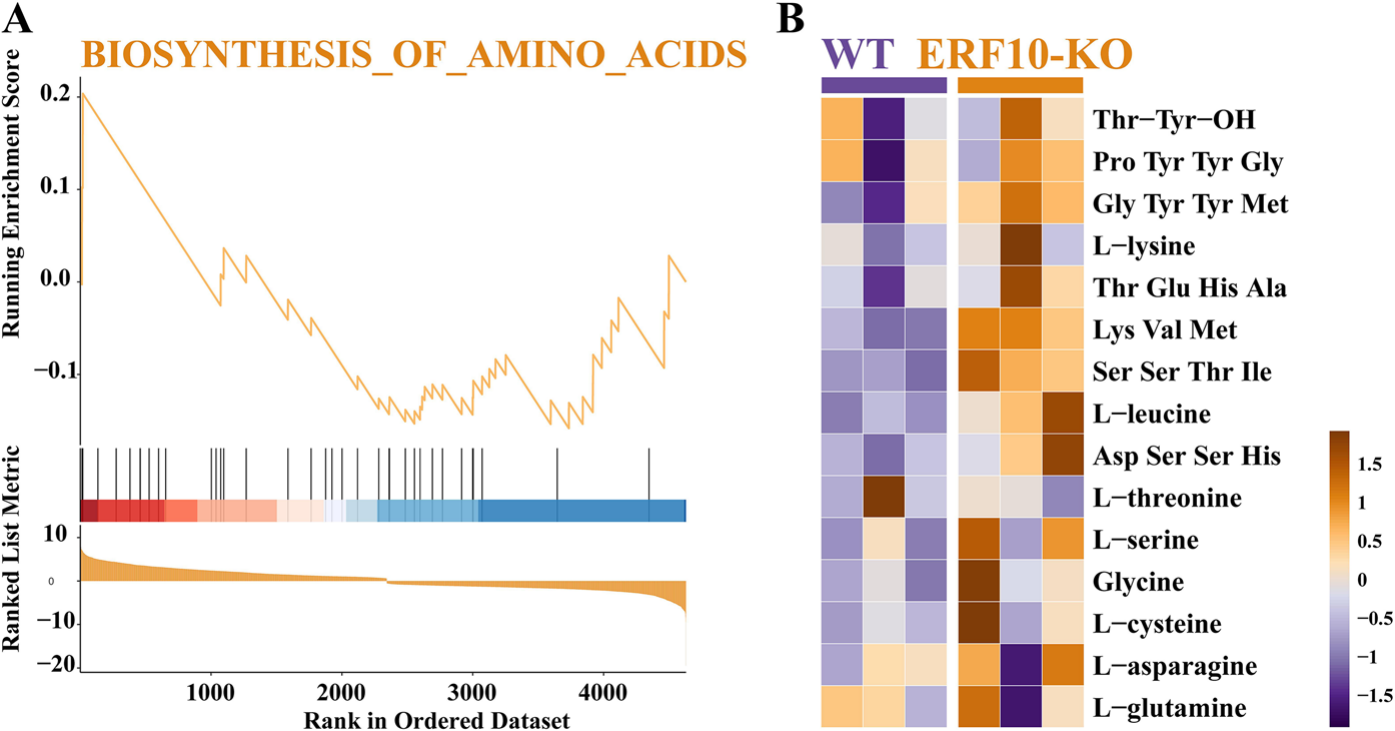
Combined functional analysis of amino acids biosynthesis genes and amino acids contents

Transcriptome and metabolome analyses were combined to determine the physiological and biochemical processes involved in differential genes and differential metabolites. The combined functional analysis of differential genes and differential metabolites showed that they were enriched in the biosynthesis of amino acids. In ERF10-KO, the expression of amino acid synthesis genes and free amino acid levels were upregulated. These results indicated that metabolism was active and catabolic in the gene-edited lines at maturity, which may be closely related to *NtERF10* knockout in tobacco (Fig. 7).

**Fig. 8:**
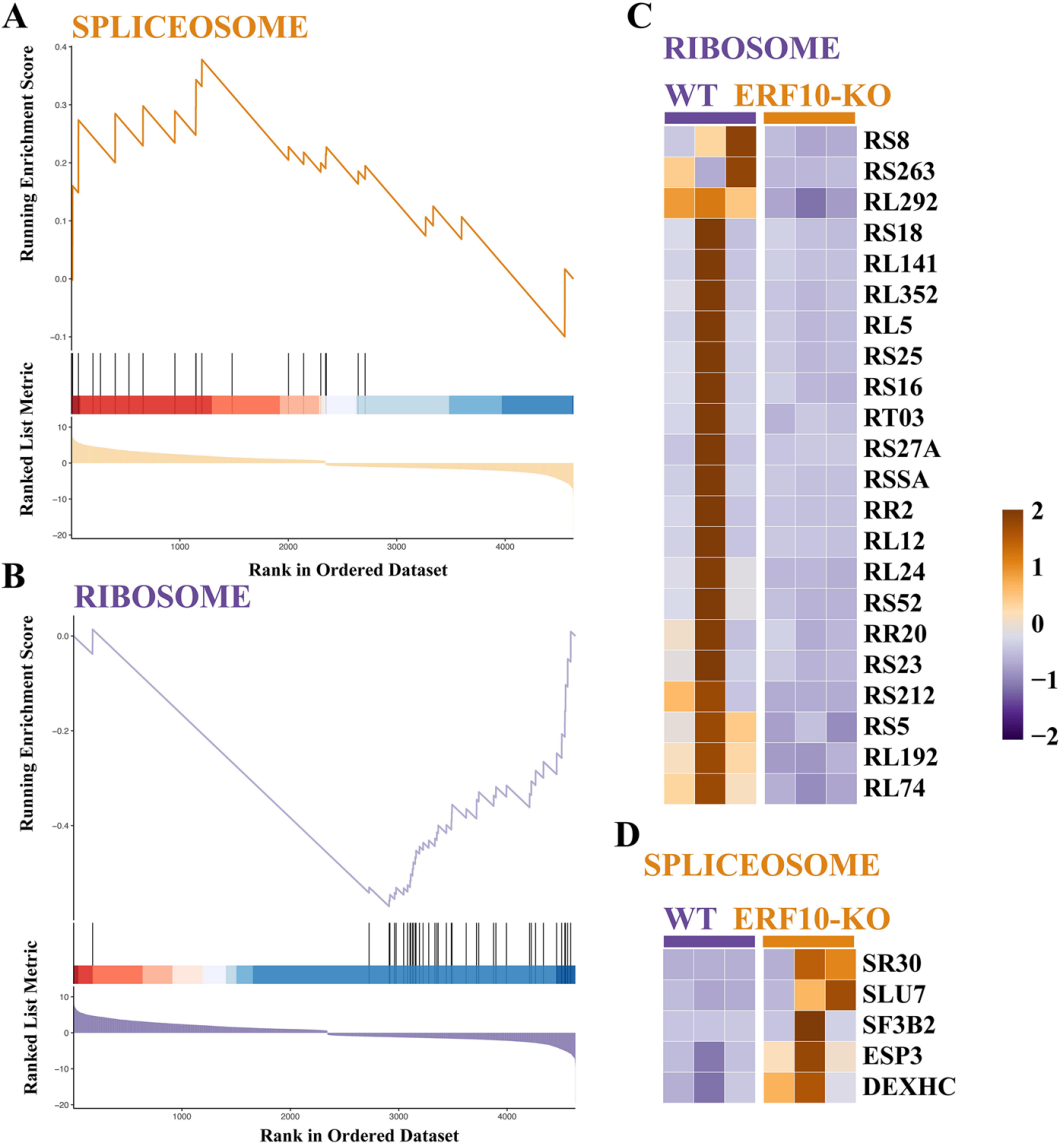
Splicing and translation-related differential gene changes in ERF10-KO and WT. **A** and **B** GSEA analysis of splicing and translation related genes. **C** and **D** Specific differential gene changes in ribosome and spliceosome

Further analysis of the physiological processes related to transcription and translation showed that the expression of the genes SR30, SLU7 and SF3B2 was upregulated in ERF10-KO compared with the WT at the transcription stage, while many genes (e.g., *RSs*, *RLs* and *RRs*) were downregulated at the translation stage (Fig. 8). These results indicated that *NtERF10* gene knockout affected the transcriptional translation stage and basal metabolism stage.

### Expression changes in dwarfing-related transcription factors

**Fig. 9:**
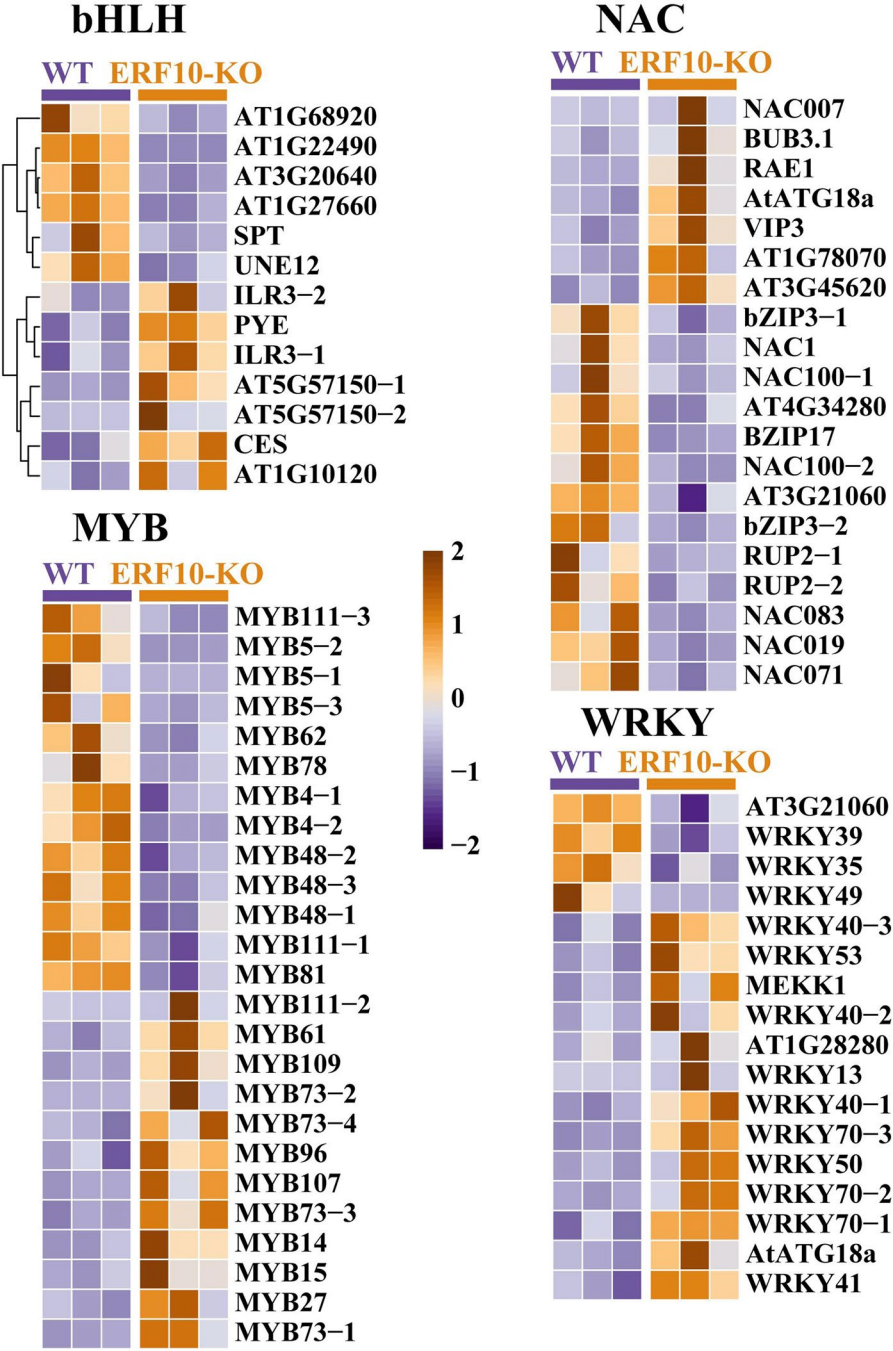
Identification and expression of transcription factors linked to dwarfism in ERF10-KO and WT. The transcription factor families bHLH, NAC, MYB, and WRKY are included. **bHLH,** basic helix-loop-helix; **NAC,** Nac protein; **MYB**, MYB proteins; **WRKY,** WRKY proteins

It is important to identify transcription factors affecting plant growth and development. Analysis of transcription factors that may affect plant dwarfing. TFs involved in growth and development showed differential expression in the mutant and wild type (Fig. 9). These TFs include members of the bHLH, NAC, MYB and WRKY TF families. In mutant plants, the expression of the transcription factors *SPT*, *NAC1*, *NAC019*, *MYB4, MYB5*, *MYB48*, *WRKY35*, *WRKY39* and *WRKY49* decreased, while the expression of the *PYE*, *NAC007*, *MYB73* and *WRKY70* genes increased. It was speculated that these transcription factors are involved in important physiological processes such as dwarfism and the growth and development of plants.

## Discussion

### Studies on the development and resistance of ERF genes in plants

*ERF* genes play an important role in regulating growth, development and resistance in plants. *OsEATB*, a rice AP2/ERF gene, restricts internode elongation, resulting in reduced plant height and panicle length (Qi et al. 2011). *TaERF8*s are differentially involved in the regulation of wheat growth and development (Zhang et al. 2020a). The GSEA of the differentially expressed gene enrichment pathways in the transcriptome (Fig. 2) showed that the differentially expressed genes were mainly enriched in photosynthesis, plant pathogen interaction, plant hormone signal transduction, amino acid metabolism and transcription and translation processes (Figs. 2, 3, 4).

The results showed that the expression levels of genes related to growth and development and stress resistance were downregulated in the ERF10-KO plants (Fig. 3). *psbA* (encoding the D1 protein) plays an important role in protecting photosystem II (PSII) from oxidative damage in higher plants. In the dwarf mutant plants, the expression levels of the *NtpsbA* gene were decreased. This result is in agreement with the findings of a previous study revealing that overexpression of *ZmpsbA* enhanced protection in biochemical components of photosynthesis (Huo et al. 2015). Cell membrane lipid metabolism-related genes, such as *DGK*s, *PLA*s and *LPP*s*,* were downregulated. *DGK*s have been found to play a crucial role in several plant abiotic stress responses, especially regarding their significant role in lipid signaling in plants (Carther et al. 2019). Studies have shown that glutathione metabolism may participate in the growth and development of animals and plants by regulating cell division and differentiation (Stasolla 2010). Our results indicated that *GPX7* gene expression was decreased in the NtERF10 knockout line, which may have affected plant growth and development. This is consistent with the findings of microarray experiments that unveiled numerous differentially expressed chloroplast-encoded genes from the gpx7 mutant compared with the WT (Li et al. 2022).

Studies in plants have been successful in identifying transcription factors that play important roles in regulating growth and development and abiotic stress responses (Fig. 4). The results in the present study showed that the expression levels of *ERF4* and *ERF9* were lower in the mutant than in the wild type. Similar results have been found in other plants; Arabidopsis overexpressing *ERF4* exhibited much larger cells and organs, while plants that lacked functional *ERF4* displayed smaller organs than the wild type (Ding et al. 2022). In the GA signaling pathway, GA2-oxidases (*GA2oxs*) are critical for the inactivation of GA, especially during vegetative growth (Thomas and Hedden 1999). *OsGA2ox5-*overexpressing rice plants exhibit dominant dwarf and GA-deficient phenotypes compared to wild-type plants (Shan et al. 2014). Overexpression of *OsRPH1* was found to reduce the plant height of rice, and the expression levels of the GA inactivation genes *OsGA2ox7*, *OsGA2ox9*, and *OsGA2ox10* were shown to be significantly upregulated in OsRPH1-OE lines compared to the WT. In contrast, the expression level of *OsGA2ox1*-*OsGA2ox3* was not clear (Ma et al. 2020). These results indicated that *GA2ox* gene expression had different effects on GA content in plants. Studies have shown that the BR-deficient cotton mutant *pagoda1* (pag1) had a smaller leaf size than the WT. The expression of the *EXORDIUM* (*GhEXO2*) gene was significantly downregulated in pag1 (Li et al. 2023). Here, we found that the expression levels of *EXO1–EXO3* were lower in the ERF10-KO than in the WT plants.

Plant terpenoids are synthesized through the MVA and MEP pathways, which are involved in plant growth and development and the stress response (Karunanithi and Zerbe 2019; Pu et al. 2021). To clarify the molecular mechanisms underlying plant height differences between the ERF10-KO and the WT plants, we analyzed the expression patterns of 70 terpene synthesis-related genes in the MVA and MEP pathways. The expression patterns of terpene synthesis genes were different in ERF10-KO and WT. Differences in the expression patterns of terpene synthesis-related genes appear to be the underlying reason for the plant height and resistance between ERF10-KO and WT. *DXS*, *HMGR*, *HDR*, and *FPPS* also play a role in the synthesis of terpene compounds in *Zanthoxylum japonica* fruit (Fei et al. 2021), and these genes are key genes for terpenoid synthesis in grape (Sun et al. 2014). *HMGR*, *FPPS*, and *TPSs* also played a role in terpenoid synthesis in tobacco, indicating that terpenoid synthesis pathways and key genes are conserved among different species.

In addition to analysis of different metabolite enrichment pathways, it was found that the pyruvate metabolism pathway and amino acid (valine, leucine and isoleucine biosynthesis) pathway and the products of acetyl-CoA were related to the terpenoid synthesis pathway.

### Integrated analysis of the transcriptome and metabolome

Metabolites are the intermediate or final products produced in the plant growth process and strongly regulate plant growth and development. mRNA sequencing can elucidate the dynamics of functional genes, reveal genome-wide genetic variations, and show which genes are closely associated with phenotypic traits. Transcriptomic analyses of such mutants can reveal candidate genes affecting phenotypic traits (Zhang and Hao 2020). During this process, it was found that the expression of amino acid biosynthesis-related genes was upregulated, the levels of free amino acids were upregulated, and the spliceosome- and ribosome-related genes were changed, which was related to growth and development. These results indicated that gene editing of *NtERF10* induced dramatic changes in substance and energy metabolism. Numerous ribosomal-protein-defective mutants show common and rare developmental phenotypes, including growth defects, changes in leaf development, and auxin-related phenotypes (Gorou et al. 2012).

### Genes and transcription factors affecting plant growth and development pathways

We analyzed the largest TF families in tobacco (Fig. 9), the bHLH, NAC, MYB, and WRKY families. These account for over one-third of the total number of tobacco TFs (Rushton et al. 2008a). bHLH transcription factors have been studied in cotton and *Arabidopsis thaliana* and have a close relationship with plant height, growth and development (Wu et al. 2021; Imai et al. 2006). Studies have reported the functions of *OsNAC2*, encoding an NAC transcription factor in rice, in rice growth and development. Transgenic plants that constitutively expressed *OsNAC2* had shorter internodes and shorter spikelets (Chen et al. 2015). *MYB*s are also important in regulating plant growth, development, metabolism and stress response, and almost all eukaryotes have MYB transcription factors (Zhang et al. 2020b)*.* Previous studies have reported double mutants of wrky46 wrky70 and wrky54 wrky70 in Arabidopsis to determine their roles in plant growth (Chen et al. 2017). Studies have shown that *NtERF10* gene editing affects transcription factor gene expression and consequently affects growth and development in tobacco.

## Conclusion

In this study, transcriptome and metabolome detection techniques were used to detect the transcripts and metabolites of dwarf mutant and wild type tobacco. The molecular mechanism of the effect of *ERF10* gene editing on plant height was analyzed. A total of 4016 DEGs were identified in the tobacco transcriptome, including 2051 upregulated and 1965 downregulated genes in the leaves. The total number of detectable differential metabolites was 128, including 19 differential metabolites that were downregulated. A combined analysis of the metabolome and transcriptome is an effective method for analyzing the relationship between key genes and metabolites in biosynthetic pathways. This study also provided valuable data and results for the physiological processes of terpenoid synthesis, amino acid metabolism, transcription and translation in tobacco (dwarfing mutants and wild types), but the specific mechanisms still need to be further studied and explored.

### Supplementary Information

Below is the link to the electronic supplementary material.

Fig. S1 Analyzed of NtERF10 protein squence. A: The conserved domain of NtERF10 protein sequence; B: Alignment of NtERF10 in relation to AP2 members from other plant species; The conserved AP2 domains were marked in red words; typical amino acid residues at the14th (V) and the 19th (E) positions of sequence were indicated by red arrow (PNG 547 kb)
