# Supplementary figures and images for: A novel AP2/ERF transcription factor, NtERF10, positively regulates plant height in tobacco
Source: Transgenic Res. 2024 Aug 6;33(4):195–210. doi: 10.1007/s11248-024-00383-z (PMC11319389; doi:10.1007/s11248-024-00383-z)

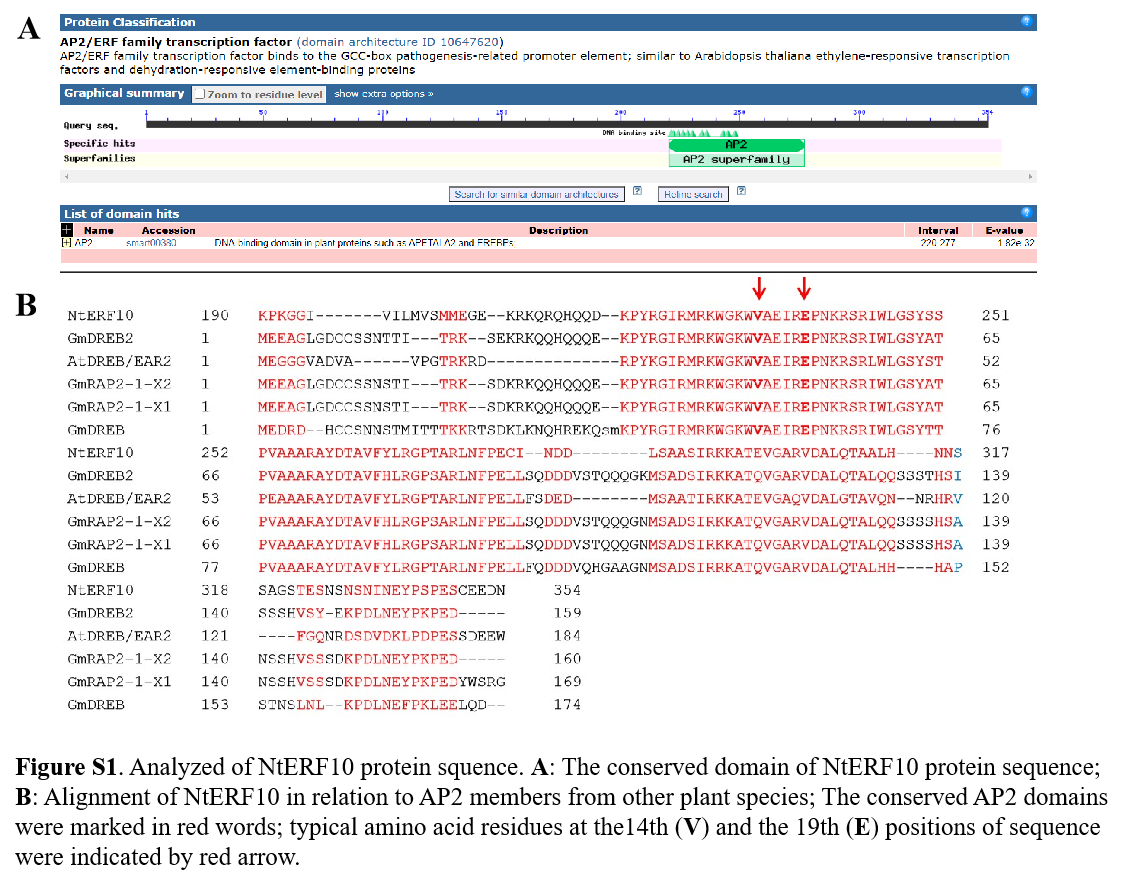

Supplement: Supplementary file 1 — Fig. S1 Analyzed of NtERF10 protein squence. A: The conserved domain of NtERF10 protein sequence; B: Alignment of NtERF10 in relation to AP2 members from other plant species; The conserved AP2 domains were marked in red words; typical amino acid residues at the14th (V) and the 19th (E) positions of sequence were indicated by red arrow (PNG 547 kb) [file 11248_2024_383_MOESM1_ESM.png]
